# Adenotonsillectomy and Neurocognitive Deficits in Children with Sleep Disordered Breathing

**DOI:** 10.1371/journal.pone.0007343

**Published:** 2009-10-06

**Authors:** Mark J. Kohler, Kurt Lushington, Cameron J. van den Heuvel, James Martin, Yvonne Pamula, Declan Kennedy

**Affiliations:** 1 Children's Research Centre, University of Adelaide, North Adelaide, Australia; 2 Centre for Sleep Research, University of South Australia, Adelaide, Australia; 3 School of Psychology, University of South Australia, Adelaide, Australia; 4 Department of Respiratory and Sleep Medicine, Women's and Children's Hospital, North Adelaide, Australia; University of Sydney, Australia

## Abstract

**Background:**

Sleep Disordered Breathing (SDB) is a common childhood disorder that encompasses a range of sleep-related upper airway obstruction. Children with SDB demonstrate significant neurocognitive deficits. Adenotonsillectomy is the first line of treatment for SDB and whilst this improves respiratory disturbance, it remains to be established whether neurocognitive gains also result.

**Methods:**

A total of 44 healthy snoring children aged 3–12 years awaiting adenotonsillectomy (SDB group), and 48 age and gender matched non-snoring controls from the general community, completed the study. All children underwent polysomnography and neurocognitive assessment at baseline and after a 6-month follow-up (after surgery in the snoring group). Our primary aim was to determine whether neurocognitive deficits in snoring children were significantly improved following adenotonsillectomy.

**Results:**

Wide ranging neurocognitive deficits were found at baseline in SDB children compared to controls, most notably a 10 point IQ difference (*P*<.001) and similar deficits in language and executive function. Whilst adenotonsillectomy improved respiratory parameters and snoring frequency at 6 months post surgery, neurocognitive performance did not improve relative to controls.

**Conclusion:**

Adenotonsillectomy successfully treated the respiratory effects of SDB in children. However, neurocognitive deficits did not improve 6-months post-operatively.

## Introduction

Sleep disordered breathing (SDB) is common in children and varies along a continuum of upper airway obstruction from primary snoring to Obstructive Sleep Apnea Syndrome (OSAS). Primary snoring is characterized by frequent snoring without ventilatory abnormalities or obvious sleep disruption and affects 5–10% of children, while the more severe OSAS is characterized by hypoxia and sleep fragmentation in 1–4% of children [1]. There is convincing evidence that even mild SDB is associated with neurocognitive deficits, particularly those of memory, learning, attention, executive functioning and cognitive capacity [2].

Historically, adenotonsillectomy has been the treatment of choice in children with SDB. While there is robust evidence that adenotonsillectomy reverses ventilatory and sleep deficits in children with upper airway obstruction [3], the consensus view is that the same applies to neurocognitive and behavioral deficits [4]. However the evidence on which this view is based is less compelling. A number of studies have reported improved attention, executive functioning, analytical thinking, verbal functioning, memory and academic progress at 6–12 months post- adenotonsillectomy [5]–[9], but others have reported no improvement in measures of language skills, visual perception, memory and executive function [6], [9], [10]. These mixed findings are limited by methodological issues which include the failure to screen children for confounding psychological disorders - particularly attention deficit hyperactivity disorder (ADHD) which is reported to co-vary with SDB, as well as a lack of controls, small subject numbers, and a restricted range of cognitive domains assessed [5], [8], [9], [11]–[13]. In addition, relatively few studies have quantified SDB using polysomnography and only one study has evaluated both SDB and control children with polysomnography at pre and post-surgical time points [5]. The latter is important as the current consensus is that many children continue to have upper airway obstruction post adenotonsillectomy [3]. Given the above limitations this study evaluated whether or not adenotonsillectomy improved both respiratory and neurocognitive function in a relatively large study of children with SDB and matched controls.

## Methods

Using a prospective repeated measures design, this study examined neurocognitive performance and severity of upper airway obstruction using polysomnography in snoring children awaiting adenotonsillectomy at baseline and six months following surgery, as compared to measures at the same time points in non-snoring control children matched for age and gender.

### Participants

Participants were recruited between November 2003 and September 2005. The study was approved by the Child, Youth and Women's Health Service Human Research Ethics Committee and families were given an AU░50 honorarium for attending each assessment trial. SDB children were those with a history of frequent snoring and who were scheduled for adenotonsillectomy because of suspected SDB by experienced pediatric otorhinolaryngologists at the Women's and Children's Hospital (WCH), North Adelaide, Australia. The sample was further restricted to children aged 3.0–12.9 years to facilitate neuropsychological testing and to avoid the potential influence of late pubertal developmental changes on sleep and upper airway dynamics [14]–[18]. Children were excluded if they spoke English as a second language, had undergone previous ENT or craniofacial surgery, had a medical or psychological condition associated with hypoxemia, sleep fragmentation, cognitive deficits and/or behavioral problems, and if they were currently taking medications known to affect sleep, respiratory dynamics or neuropsychological measures such as a stimulant or psychotropic drugs. The non-snoring control children were recruited through the recommendation of participating parents of snoring children, and from advertisements in local newspapers and schools. Similar exclusion criteria were applied to controls with the addition that they did not snore more than two nights per week as confirmed by parental report. Information regarding the age of onset and duration of snoring was collected from parents.

Socioeconomic status (SES) was determined using the Australian Bureau of Statistics' Index of Relative Socio-economic Advantage/Disadvantage 2006 national census data. A higher score on this index indicates increased income and occupational skills and/or training within the geographical area of residence (collection district), with a national population mean of 1000 and standard deviation of 100. Height and weight were measured on the polysomnographic nights and established growth charts, corrected for age and gender, were used to determine body mass index (BMI) [19].

Neurocognitive assessment at both baseline and follow-up were performed 0.9 (2.9) weeks before polysomnography (1.0 (2.7) for controls and 0.7 (3.1) for SDB children). The mean (SD) duration between baseline and follow-up polysomnography was 29.4 (5.9) weeks (27.4 (4.8) for controls and 31.5 (6.3) for SDB children) and for neurocognitive assessment was 30.8 (6.2) weeks (29.5 (3.4) for controls and 31.6 (7.7) for SDB children). The mean (SD) duration between adenotonsillectomy and follow-up polysomnography for SDB children was 27.5 (6.0) weeks and for neurocognitive assessment was 26.9 (5.8) weeks. All group differences are non-significant.

### Neurocognitive assessment

The following neurocognitive tests were administered: the Stanford Binet Intelligence Scale 5^th^ edition [20] and a Neuropsychological Developmental Assessment (NEPSY) [21]. Both tests are normed and well-validated instruments with robust validity and reliability. The Stanford Binet provides measures of intellectual capacity (Verbal and Non-Verbal IQ and composite IQ), Fluid Reasoning (FR) (inductive and deductive reasoning), Knowledge (KN) (general information), Quantitative Reasoning (QR) (numerical ability), Visual-Spatial processing (VS) (ability to process spatial information) and Working Memory (WM) (capacity to use short term memory in problem solving). The NEPSY was used to obtain the following measures of auditory and visual attention, planning and problem solving, inhibition, language development, sensorimotor function and memory and learning. A psychologist blinded to child status administered the tests during a single session on a weekday.

### Polysomnography

The Compumedics S-Series Sleep System (Melbourne, Australia) was used to collect electroencephalograhic (EEG; C3-A2 or C4-A1), left and right electrooculograhic (EOG), sub-mental and diaphragmatic electromyographic (EMG) data. Leg movement was assessed by piezoelectric motion detection, heart rate by electrocardiogram (ECG), oro-nasal airflow by thermistor and nasal pressure, respiratory movements of the chest and abdominal wall using uncalibrated respiratory inductive plethysmography (RIP), arterial oxygen saturation (SaO_2_) by pulse oximetry (Nellcor N-595; 2 second averaging time) and transcutaneous CO_2_ (TcCO_2_) using a heated (41°C) transcutaneous electrode (TINA, Radiometer Pacific). All data was digitized and stored on computer disk for subsequent analysis. Children were continuously monitored via infrared camera by a pediatric sleep technician who also documented observations of sleep behavior, which included the presence or absence of snoring.

### Data Analysis

An experienced sleep technician blinded to child status scored the studies according to standardized sleep stage [22] and pediatric ventilatory criteria [23]. Stage 3 and 4 sleep were combined for analysis as slow wave sleep (SWS). All respiratory events were scored if ≥2 respiratory cycles in duration and associated with a minimum 3% SaO_2_ desaturation and/or an arousal within two breaths of event termination. Obstructive apneas were defined as the absence of airflow associated with continued chest and abdominal wall movement. Obstructive hypopneas were defined as a ≥50% reduction in the amplitude of RIP and/or airflow signal associated with paradoxical chest/abdominal wall movement. The presence of any other supportive data such as increased diaphragmatic or submental EMG activity was further used to distinguish obstructive from central hypopneas. Central apneas were scored if there was an absence of respiratory effort as determined by RIP and diaphragmatic EMG in association with an absence of airflow. Central apneas were also scored if the event lasted ≥20 seconds. Central hypopneas were defined as a ≥50% reduction in airflow from baseline in association with a ≥50% reduction in respiratory effort from baseline. Apnea events that included both central and obstructive components were scored as a mixed apnea. The obstructive apnea/hypopnea index (OAHI) was calculated as the total number of obstructive apneas, mixed apneas and obstructive hypopneas per hour of total sleep time. An OAHI ≥1 was considered indicative of OSAS. The central apnea/hypopnea index (CAHI) was calculated as the total number of central apneas and central hypopneas per hour of total sleep time.

Spontaneous and respiratory cortical arousals were scored according to the criteria of the American Sleep Disorders Task Force [24]. Spontaneous arousal index (SAI) was expressed as the total number of spontaneous arousals per hour of total sleep time and respiratory arousal index (RAI) as the total number of respiratory arousals per hour of total sleep time. Periodic Limb Movements (PLM) were scored using standard criteria [25]. The PLM index (PLMI) was defined as the number of PLM per hour of total sleep time.

### Statistical Analysis

Independent samples t-tests were used to compare continuous demographic data, while Chi-square or Mann-Whitney tests were used to determine group differences in ordinal and nominal demographic data. A one within groups (assessment time) and one between groups (group) repeated measures analysis of covariance (ANCOVA), co-varying for significant group differences in demographic variables, was used to assess the effect of time and subject status on neurocognitive performance. Effect sizes were determined using partial eta squared values (η_p_2). Apart from PLMI, RAI, frequency of SaO_2_ desaturations ≥3%/hr total sleep time (TST), percentage of sleep time with SaO_2_ <95%, TcCO_2_ >50 mmHg, OAHI and CAHI, all PSG variables were normally distributed. An inverse transformation [1/(x+1)] was used to correct skew and transformed values were used in analyses. Non-transformed values are reported in the tables. Student t-tests were used for post-hoc testing of group differences. All p values are 2-tailed, with statistical significance determined at α = .05. Data are presented as mean (SD) unless otherwise stated.

## Results

Two hundred and twenty-six parents of snoring children awaiting adenotonsillectomy for clinically suspected OSAS were approached as potential participants. Of 81 children whose parents expressed an interest, 21 children failed to meet inclusion criteria, 6 underwent surgery before the commencement of the study, and 10 failed to complete PSG or neuropsychological assessment at baseline or follow-up resulting in a final total of 44 SDB children scheduled for adenotonsillectomy with complete baseline and post surgery data. Of 61 control non-snoring children whose parents expressed an interest, 8 failed to meet inclusion criteria, 4 failed to complete PSG or neuropsychological assessment at baseline or follow-up, and 1 was excluded because they had clinically significant OSAS at baseline (i.e. OAHI >1) resulting in a final total of 48 control children. All children had normal hearing as determined by a trained audiologist using air conduction testing and bone conduction and tympanometry assessment.

Compared to controls the SDB children, had significantly greater body mass, and significantly lower SES, although average ratings for each group were within the normal range. Group differences in age approached statistical significance. Child's age, BMI z-score and SES were therefore entered as covariates in subsequent analyses (Table 1).

**Table 1:** Demographic characteristics of control and adenotonsillectomy children.

Demographic	Control	Adenotonsillectomy
	(n = 48)	(n = 44)
Age, years	7.7 (2.6)	6.6 (2.6), *p* = 0.05
Gender, n males (%)	22 (45.8%)	15 (62.5%)
Ethnicity, n (%)		
Caucasian	48 (100%)	42 (95.4%)
Asian	0 (0%)	1 (2.3%)
Hispanic	0 (0%)	1 (2.3%)
BMI z-score	0.29 (0.9)	0.84 (1.3)*
SES	1006.7 (88.1)	941.2 (110.5)†
Snoring duration, years	0 (0)	3.6 (2.8)‡
Smoking in home, n yes (%)	13 (27.1%)	16 (36.4%)
Parental history, n yes (%)		
Snoring	30 (62.5%)	31 (70.5%)
Sleep Apnea	7 (14.6%)	7 (15.9%)
Adenoidectomy and/or tonsillectomy	21 (43.8%)	25 (56.8%)

*denotes *p*<0.05, †*p*<0.005 and ‡*p*<0.001.

**Figure 1:**
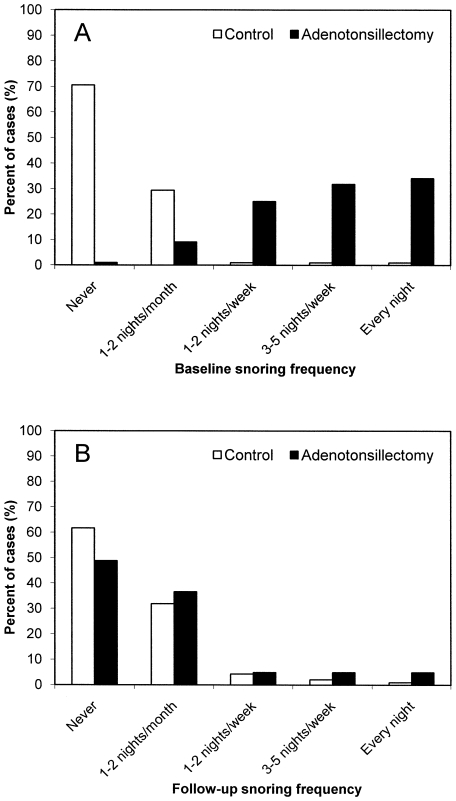
Parentally reported snoring frequency for controls and children awaiting adenotonsillectomy. Children scheduled for adenotonsillectomy were reported by parents to snore more frequently compared to controls, χ^2^ = 79.7, *p*<.001 (Figure 1A). At 6 months follow-up, snoring frequency was not significantly greater amongst children who underwent adenotonsillectomy compared to controls, χ^2^ = 3.8, *p* = 0.433 (Figure 1B).

Compared to baseline (Figure 1a), snoring frequency was significantly lower at follow-up in the SDB group, (p<.001, Wilcoxon Signed Ranks test) where it reached a similar frequency to that of controls (Figure 1b).

### Polysomnography

The mean and standard deviation PSG values at baseline and follow-up are presented in Table 2. Across both time points, SDB children had significantly higher SWS and less stage 2 sleep compared to control children. No other group differences were found for measures of sleep architecture. Respiratory arousals were significantly elevated at baseline in SDB compared to control children but were not different to controls post-surgery. Group differences were not found for frequency of spontaneous arousals. SDB children had a significantly greater frequency of SaO_2_ desaturations ≥3% and a greater proportion of sleep with SaO_2_ below 95% compared to control children across both time points. Despite this, the range in frequency of desaturation was markedly reduced following treatment in the SDB group (from 0–53.1 to 0–5.6). No significant differences were observed for TcCO_2_. Both OAHI and CAHI were significantly greater for SDB children compared to controls at baseline. Adenotonsillectomy improved upper airway obstruction to levels equivalent to controls, with a significant mean pre- to post-operative reduction in OAHI of 5.8 to 0.8.

**Table 2:** Polysomnography results for control and adenotonsillectomy children during baseline and follow-up assessments.

	Baseline		Follow-up		F-value		
	Control	Adenotonsillectomy	Control	Adenotonsillectomy	Group	Time	Group × Time
Total Sleep Time (min)	447.0 (35.2)	436.7 (49.3)	451.8 (54.0)	449.1 (54.1)	0.62	2.16	0.42
Stage 1, % of TST	3.3 (1.8)	2.8 (2.0)	3.1 (2.3)	3.0 (2.4)	0.30	0.00	0.75
Stage 2, % of TST	44.6 (5.9)	42.4 (5.4)	46.7 (5.1)	42.7 (7.0)	8.49**	3.25	1.85
SWS, % of TST	31.7 (5.6)	34.4 (6.2)	30.0 (4.9)	33.7 (6.0)	10.21†	3.56	0.54
REM, % of TST	20.4 (4.1)	20.3 (5.3)	20.3 (4.2)	20.6 (4.1)	0.01	0.00	0.14
REM latency (min)	91.8 (21.4)	89.9 (29.0)	92.5 (25.4)	93.3 (24.3)	0.02	0.42	0.17
Movement time (min)	9.3 (4.8)	9.2 (4.3)	9.5 (4.8)	8.7 (4.0)	0.33	0.08	0.66
WASO (min)	40.2 (28.7)	45.3 (40.1)	39.4 (36.7)	52.9 (45.5)	2.34	0.43	0.67
Awakenings/hour TST	0.78 (0.5)	0.79 (0.7)	0.67 (0.6)	0.69 (0.5)	0.02	3.03	0.02
Stage shifts/hour TST	12.2 (2.8)	12.9 (2.8)	12.1 (2.9)	12.8 (3.2)	2.01	0.13	0.00
PLMI^1^, median (range)	1.4 (0–22.6)	1.7 (0–24.7)	0.8 (0–11.9)	2.3 (0–26.4)	1.25	1.67	0.20
SAI	9.4 (2.9)	8.5 (2.6)	9.4 (2.6)	9.4 (3.2)	0.73	2.26	2.95
RAI^1^, median (range)	0.4 (0–2.5)	1.2 (0–33.9)	0.4 (0–2.4)	0.7 (0–9.6)	22.04‡	5.33*	10.95†
SaO_2_ desats ≥3%/hr TST^1^, median (range)	0.7 (0–4.9)	1.4 (0–53.1)	0.6 (0–3.0)	1.5 (0–5.6)	22.16‡	2.34	0.54
SaO_2_ <95%, % TST^1^, median (range)	0.1 (0–48.5)	0.9 (0–64.0)	0.1 (0–19.9)	0.3 (0–53.0)	16.00‡	0.05	1.36
TcCO_2_ >50 mmHg, % TST^1^, median (range)	18.3 (0–70.2)	21.2 (0–83.8)	19.6 (0–67.6)	5.4 (0–69.9)	0.09	2.20	0.05
OAHI^1^, median (range)	0.13 (0–1.0)	0.78 (0–49.8)	0.15 (0–2.5)	0.36 (0–4.7)	30.73‡	4.69*	23.08‡
OSAS, n (% OAHI ≥1)	0 (0)	20 (44.5)	4 (8.3)	15 (34.0)			
CAHI^1^, median (range)	0.41 (0–4.4)	0.66 (0–13.5)	0.39 (0–2.8)	0.80 (0–4.1)	8.21**	1.11	0.30

TST  =  total sleep time; SWS  =  slow wave sleep; REM  =  rapid eye movement sleep; WASO  =  wake time after sleep onset; PLMI  =  periodic limb movement index; SAI  =  spontaneous arousal index; RAI  =  respiratory arousal index; OAHI  =  obstructive apnea/hypopnea index; CAHI  =  central apnea/hypopnea index. ^1^Analysis performed using transformed values. *denotes *p*<0.05, ** *p*<0.01, †*p*<0.005 and ‡*p*<0.001.

### Neurocognitive performance

The mean and standard deviation Stanford Binet and NEPSY composite and subtest values and F-test results are reported in tables 3 and 4 respectively.

**Table 3:** Comparisons across assessments and between groups for the Stanford Binet domain and subtest scores.

	Baseline		Follow-up		F-value (effect size, η_p_^2^)					
	Control	Adenotonsillectomy	Control	Adenotonsillectomy	Group		Time		Group × Time	
Full Scale IQ	110.3 (11.4)	99.8 (11.2)	111.6 (11.4)	101.3 (10.7)	31.09‡	(0.26)	0.41	(0.01)	0.13	(<0.01)
Fluid Reasoning	110.7 (14.3)	100.1 (14.8)	110.4 (13.2)	100.3 (12.3)	16.84‡	(0.16)	0.08	(<0.01)	0.04	(<0.01)
Knowledge	103.8 (9.9)	94.1 (10.4)	105.9 (10.6)	96.4 (11.5)	28.36‡	(0.25)	0.06	(<0.01)	0.17	(<0.01)
Quantitative Reasoning	109.1 (13.9)	104.9 (13.0)	110.2 (13.5)	103.1 (12.8)	10.78†	(0.11)	<0.01	(<0.01)	1.13	(0.01)
Visual Spatial	109.5 (11.8)	97.8 (13.9)	108.6 (12.1)	102.1 (12.2)	19.73‡	(0.19)	0.24	(<0.01)	4.84*	(0.05)
Working Memory	112.0 (14.3)	103.2 (12.2)	115.8 (13.3)	104.8 (11.5)	25.70‡	(0.23)	3.50	(0.04)	0.82	(0.01)
Non-Verbal IQ	109.3 (12.6)	100.0 (13.1)	111.3 (13.4)	102.7 (11.2)	22.11‡	(0.20)	0.98	(0.01)	<0.01	(<0.01)
Non-Verbal Fluid Reasoning	11.2 (3.2)	9.5 (3.5)	11.6 (2.8)	9.7 (3.2)	14.75‡	(0.15)	1.05	(0.01)	0.32	(<0.01)
Non-Verbal Knowledge	10.9 (2.6)	9.3 (2.6)	11.3 (2.7)	9.6 (2.1)	11.03†	(0.11)	0.01	(<0.01)	0.06	(<0.01)
Non-Verbal Quantitative Reasoning	11.7 (2.7)	11.5 (2.8)	11.8 (2.5)	10.9 (2.3)	6.38*	(0.07)	0.30	(<0.01)	2.11	(0.02)
Non-Verbal Visual Spatial	11.4 (2.4)	9.1 (2.9)	11.3 (2.4)	9.9 (2.7)	15.13‡	(0.15)	0.34	(<0.01)	2.10	(0.02)
Non-Verbal Working Memory	12.1 (3.2)	10.7 (2.9)	12.8 (3.3)	11.4 (2.5)	14.33‡	(0.14)	0.89	(0.01)	<0.01	(<0.01)
Verbal IQ	110.6 (10.7)	99.7 (11.2)	111.2 (11.5)	100.2 (13.2)	30.30‡	(0.26)	<0.01	(<0.01)	0.26	(<0.01)
Verbal Fluid Reasoning	12.4 (2.5)	10.5 (3.3)	12.0 (2.6)	10.7 (2.4)	7.97**	(0.08)	1.06	(0.01)	1.24	(0.01)
Verbal Knowledge	10.5 (1.9)	8.6 (2.0)	10.9 (1.9)	9.0 (2.9)	32.69‡	(0.27)	0.08	(<0.01)	0.32	(<0.01)
Verbal Quantitative Reasoning	11.7 (2.9)	10.3 (2.6)	12.1 (2.8)	10.3 (2.4)	14.76‡	(0.15)	0.01	(<0.01)	0.53	(0.01)
Verbal Visual Spatial	12.0 (2.8)	10.2 (3.0)	11.9 (2.4)	10.8 (2.2)	15.24‡	(0.15)	0.96	(0.01)	2.02	(0.02)
Verbal Working Memory	12.0 (2.7)	10.4 (2.6)	12.8 (2.1)	10.3 (2.4)	24.71‡	(0.22)	4.52*	(0.05)	3.08	(0.03)

*denotes *p*<0.05, ** *p*<0.01, †*p*<0.005 and ‡*p*<0.001; Effect size (η_p_^2^) of 0.01, 0.06 and 0.14 =  small, medium and large effect sizes respectfully.

**Table 4:** Comparisons across assessments and between groups for the NEPSY domain and subtest scores.

	Baseline				Follow-up		F-value (effect size, η_p_^2^)					
	Control	n	Adenotonsillectomy	n	Control	Adenotonsillectomy	Group		Time		Group × Time	
Attention/Executive	114.9 (10.6)	46	101.8 (14.2)	43	118.9 (11.9)	106.5 (14.7)	28.13‡	(0.25)	0.49	(0.01)	0.32	(<0.01)
Planning	13.6 (1.9)	38	10.8 (3.6)	25	14.1 (2.2)	12.6 (2.5)	17.98‡	(0.24)	2.13	(0.04)	3.07	(0.05)
Inhibition	9.5 (2.3)	38	8.6 (2.4)	25	9.3 (1.9)	8.6 (1.8)	4.29*	(0.07)	0.18	(<0.01)	0.12	(<0.01)
Auditory Attention	10.3 (2.4)	38	9.0 (3.0)	25	10.0 (2.0)	8.9 (2.2)	5.39*	(0.09)	0.89	(0.02)	0.60	(0.01)
Visual Attention	12.3 (2.4)	46	10.9 (2.6)	43	13.7 (2.5)	11.4 (3.1)	13.93‡	(0.14)	0.14	(<0.01)	0.46	(0.01)
Language	112.4 (16.5)	46	95.7 (15.4)	43	115.6 (18.2)	98.5 (15.3)	32.86‡	(0.28)	0.78	(0.01)	0.24	(<0.01)
Phonological processing	11.7 (3.4)	46	8.1 (3.6)	43	12.2 (3.3)	9.3 (2.6)	32.83‡	(0.28)	0.37	(0.05)	0.55	(0.01)
Speeded naming	10.5 (2.7)	38	8.3 (2.6)	25	11.0 (3.3)	9.8 (2.2)	9.77†	(0.14)	0.01	(<0.01)	1.78	(0.03)
Comprehension	12.7 (2.3)	38	10.6 (3.1)	43	13.0 (2.6)	10.8 (2.8)	20.33‡	(0.20)	2.97	(0.09)	0.78	(0.01)
Sensorimotor	98.8 (13.6)	46	92.1 (14.1)	41	101.4 (13.9)	96.7 (13.5)	11.39†	(0.12)	4.82*	(0.06)	0.05	(<0.01)
Finger tapping	9.7 (2.9)	38	8.2 (2.3)	25	10.4 (2.6)	10.0 (1.7)	3.01	(0.05)	0.24	(0.02)	2.51	(0.04)
Imitating hand positions	10.2 (2.9)	46	9.0 (2.7)	41	11.2 (2.8)	10.2 (2.6)	8.37**	(0.09)	1.08	(0.01)	0.13	(<0.01)
Visuomotor function	9.8 (2.8)	46	9.2 (2.9)	43	9.0 (2.9)	8.7 (3.3)	3.60	(0.04)	2.00	(0.02)	0.03	(<0.01)
Memory	108.0 (15.6)	46	99.6 (15.0)	43	117.5 (12.1)	107.1 (15.6)	7.75**	(0.08)	2.96	(0.03)	0.92	(0.01)
Faces	11.2 (2.9)	38	11.9 (2.3)	25	13.9 (2.5)	14.3 (2.9)	0.29	(0.01)	10.16†	(0.15)	1.44	(0.02)
Names	10.6 (3.4)	38	9.4 (3.4)	25	12.5 (3.0)	11.4 (3.0)	1.43	(0.02)	10.11†	(0.15)	0.51	(0.01)
Narrative	11.4 (2.8)	46	9.1 (2.9)	43	11.6 (2.0)	9.8 (2.6)	16.88‡	(0.17)	0.16	(<0.01)	0.88	(0.01)

*denotes *p*<0.05, ** *p*<0.01, †*p*<0.005 and ‡*p*<0.001; Effect size (η_p_^2^) of 0.01, 0.06 and 0.14 =  small, medium and large effect sizes respectfully.

#### Stanford Binet

The composite Verbal, Nonverbal and Full Scale IQ were significantly lower at preoperative baseline in SDB compared to control children; however scores for all children were on average within the standardised normal range. The magnitude of this deficit persisted at six month follow-up with a mean Full Scale IQ difference of 10 points between control and SDB children.

The Fluid Reasoning, Knowledge, Quantitative Reasoning, Visual Spatial and Working Memory composite scores, and corresponding Verbal and Nonverbal subtest scores, were all significantly reduced in SDB compared to control children at both baseline and follow up. In general, effects were greatest for the verbal component of tests, and specifically for measures of Knowledge and Working Memory. A significant interaction effect was observed for the composite Visual Spatial score, due to a larger improvement in SDB children from baseline to follow-up. Nonetheless, visual spatial scores were still significantly lower in SDB children at follow-up. Observed power for significant group differences across the Stanford Binet scales were high, ranging from 0.80 to 1.0 (median  = 0.98).

#### NEPSY

Mean NEPSY composite and subtest scores for SDB children were within the standardised normal range both preoperatively and post adenotonsillectomy. Despite this, composite scores for Attention/Executive functioning, Language development, Sensorimotor function and Memory were significantly reduced across both time points in SDB children compared to controls.

Analysis of the individual subtests contributing to the composite Attention/Executive score, both at baseline and follow-up, indicated that SDB children had significantly reduced planning, inhibition, auditory and visual attention scores compared to controls. Effects were greatest for measures of planning and visual attention. Likewise composite Language subtest scores indicate both at baseline and follow-up that SDB compared to control children had significantly reduced phonological processing, comprehension and speeded naming ability. Effects were particularly large for phonological processing.

Of Sensorimotor subtests, only imitation of hand positions was reduced in SDB children compared to controls. No interaction over time was observed, however, Sensorimotor performance in general improved in both groups at follow-up.

Analysis of subtest scores contributing to the composite Memory score show that only narrative memory scores were significantly reduced in SDB children compared to controls, both at baseline and post-operatively. For all children, memory for names and memory for faces improved at follow-up. Observed power for significant group differences across the NEPSY were moderate to high, ranging from 0.53 to 1.0 (median = 0.96).

Further analyses of SDB children found no differences in neurocognitive performance on either the Stanford Binet or NEPSY when comparing those demonstrating an OAHI ≥1 vs. an OAHI <1 post-operatively.

#### Association between OAHI severity and neurocognitive performance

To investigate the association between OAHI severity and neurocognitive performance correlation analyses were performed between ventilatory (OAHI, O_2_ <95%, SaO_2_ desats ≥3%/hr TST, SaO_2_ nadir, and RAI, snoring duration) and baseline neurocognitive scores. Neurocognitive scores did not show significant associations with any of the ventilatory parameters.

The possible contribution of concurrent upper airway obstruction to neurocognitive function was further explored using a series of hierarchical multiple regression analyses. Age, SES and BMI z-score were entered in the first step and OAHI in the second step of analyses.

Baseline OAHI was not significantly predictive of baseline Stanford Binet or NEPSY scores at step 2 (β range  = 0.001 to 0.255), with the exception of verbal quantitative reasoning (β = 0.369, *p*<.01), for which OAHI explained 12.5% of the variance over and above age, BMI z-score and SES. Similarly, post-operative OAHI was not significantly predictive of post-operative Stanford Binet or NEPSY scores. When entered at the second step in place of OAHI, the change in OAHI between time points was not significantly predictive of any neurocognitive parameter.

## Discussion

The crucial findings of the present study were that in children with SDB adenotonsillectomy improved sleep and ventilatory parameters as expected but not neurocognitive performance six months post-surgery. Unlike previous studies reporting improved neurocognition, the present study excluded children with confounding psychological disorders, such as ADHD. In addition, PSG was performed in both SDB and control children at both baseline and follow-up. This is the first such study to objectively determine sleep and ventilatory parameters, and a comprehensive range of neurocognitive parameters both before and after adenotonsillectomy in SDB and control children. Consistent with previous studies, we confirmed that children with SDB have wide ranging neurocognitive deficits [2], [26]. Contrary to expectations however [5]–[12] these deficits persisted despite successful treatment of the underlying upper airway obstruction. Given the prevalence of childhood SDB, the implications of the present study's findings for daytime function and academic performance for these children are concerning.

An important finding was that the magnitude of neurocognitive deficits did not relate to the severity of upper airway obstruction. Moreover, there was no association between the improvement in sleep respiratory parameters post-adenotonsillectomy and any change in neurocognitive function. SDB children compared to matched control children had a persistent deficit at baseline and six months post adenotonsillectomy of, on average, 10 IQ points. Performance in control children was consistent with that of healthy children in other large recent studies [27]. Taken at face value the lack of association between SDB severity and neurocognitive performance implies that relatively mild SDB and the concomitant sleep fragmentation may be more harmful than is currently believed, a suggestion originally outlined in a previous study by our group [28]. Further support that even mild upper airway obstruction has detrimental neurocognitive consequences is provided by the study of O'Brien et al. [29], who reported visual attention, language, phonological processing and visuospatial deficits in 87 children with primary snoring (AHI <1) compared to controls. In addition, Giordani et al. [26] found that snoring children scheduled for adenotonsillectomy, whose PSG was normal (i.e. confirmed as primary snorers), had decrements in visual spatial problem solving, memory and arithmetic achievement comparable to those with OSAS as confirmed by PSG. Interestingly, in the latter study those children with primary snoring, *but not those with OSAS*, scored significantly lower on reading and verbal-based academic achievement, short term attention, working memory and sustained attention compared to non-snoring control children. The lack of significant correlations in this and other studies between the PSG parameters and neurocognitive functioning may be due to the limitations inherent in the PSG parameters currently recorded in children, especially their insensitivity to more subtle markers of sleep fragmentation such as sub-cortical arousals which are not routinely quantified. More refined measures of arousal such as those obtained using spectral analysis and other quantitative EEG measures may provide the answer. A relationship with current upper airway obstruction severity and neurocognitive performance may not be observed, as deficits in snoring children may be longstanding and/or cumulative.

The failure to find an improvement in neurocognitive performance at six months post-adenotonsillectomy presents a challenge to our current understanding of the relationship between upper airway obstruction and neurocognitive deficits. It may be that a longer period of post-operative recovery is required before improvements are noticeable. However, the majority of relevant studies examining children's neurocognitive performance report significant gains within 3–12 months post-adenotonsillectomy [5], [6], [8]–[13], [30]–[32]. Indeed, the results from this study do report gains in working memory function, memory for names and faces, and overall sensorimotor ability in snoring children, but these were no greater than gains observed in controls, which is consistent with a learning effect. The duration and/or age of snoring onset may also be important. However, we did not find a significant relationship between parentally reported snoring duration and any neurocognitive measure in our adenotonsillectomy group. Additional analyses, not reported here, divided the sample into three age groups (3–4 years, 5–7 years, and 8–12 years) to determine whether group differences varied by age yet did not find significant effects.

In the current study it is interesting to note the deficits showing largest effects were for executive function and verbal and language performance, in particular phonological processing. Language skills are thought to underpin much of higher learning [33], [34] and phonological processing is reported to be a good predictor of later reading ability and auditory processing [35]. A child who is unable to plan and strategize, and whose language skills are impaired is at a distinct disadvantage in classrooms in Western countries where so much of children's academic progress is dependent on verbal modes of learning and reasoning.

The results of this study are compelling and concerning, but it is important to emphasize this was not a randomized study as snoring children were identified prior to participation. Assumption of cause and effect must be made with caution until large-scale randomized controlled trials can be conducted. A multicenter randomized controlled clinical trial, to assess the impact of early intervention with adenotonsillectomy versus watchful waiting and supportive care on neurocognitive function in children aged 5 to 10 years, with OSA and adenotonsillar hypertrophy, is currently planned in the USA [36]. However, the results of the current study suggest that the genesis of long-term adverse neurocognitive effects in snoring children may be during a critical developmental period, at or before 3 years of age, so future research should include children in this age range.

In summary, adenotonsillectomy improved ventilatory parameters and snoring but neurocognitive deficits were not improved six months post-surgery. The most prominent deficits included higher cognitive functions and particularly demonstration of knowledge, executive functions of planning and working memory, as well as measures of language development such as phonological processing and comprehension. No dose response was observed between the severity of upper airway obstruction and neurocognitive deficits. Recent research has postulated that an individuals' systemic inflammatory response to hypoxia may explain differential outcomes to upper airway obstruction [37]–[39]. However, alternative and more subtle measures of arousal and sleep fragmentation may also be important mediators of neurocognitive deficits in children. It is estimated that approximately 5–10% of children snore most nights, while 1–4% of children have OSAS [1] affecting a possible 7.4 million US, 1.5 million UK and 400,000 Australian children. Given the high prevalence, the public health significance of our findings is profound and requires urgent further study in younger children.
